# Freshwater Recirculating Aquaculture System Operations Drive Biofilter Bacterial Community Shifts around a Stable Nitrifying Consortium of Ammonia-Oxidizing *Archaea* and Comammox *Nitrospira*

**DOI:** 10.3389/fmicb.2017.00101

**Published:** 2017-01-30

**Authors:** Ryan P. Bartelme, Sandra L. McLellan, Ryan J. Newton

**Affiliations:** School of Freshwater Sciences, University of Wisconsin-MilwaukeeMilwaukee, WI, USA

**Keywords:** recirculating aquaculture system, biofilter, nitrifiers, ammonia-oxidizing archaea, comammox, microbial communities, Nitrospira

## Abstract

Recirculating aquaculture systems (RAS) are unique engineered ecosystems that minimize environmental perturbation by reducing nutrient pollution discharge. RAS typically employ a biofilter to control ammonia levels produced as a byproduct of fish protein catabolism. *Nitrosomonas* (ammonia-oxidizing), *Nitrospira*, and *Nitrobacter* (nitrite-oxidizing) species are thought to be the primary nitrifiers present in RAS biofilters. We explored this assertion by characterizing the biofilter bacterial and archaeal community of a commercial scale freshwater RAS that has been in operation for >15 years. We found the biofilter community harbored a diverse array of bacterial taxa (>1000 genus-level taxon assignments) dominated by *Chitinophagaceae* (~12%) and *Acidobacteria* (~9%). The bacterial community exhibited significant composition shifts with changes in biofilter depth and in conjunction with operational changes across a fish rearing cycle. *Archaea* also were abundant, and were comprised solely of a low diversity assemblage of *Thaumarchaeota* (>95%), thought to be ammonia-oxidizing archaea (AOA) from the presence of AOA ammonia monooxygenase genes. *Nitrosomonas* were present at all depths and time points. However, their abundance was >3 orders of magnitude less than AOA and exhibited significant depth-time variability not observed for AOA. Phylogenetic analysis of the nitrite oxidoreductase beta subunit (*nxrB*) gene indicated two distinct *Nitrospira* populations were present, while *Nitrobacter* were not detected. Subsequent identification of *Nitrospira* ammonia monooxygenase alpha subunit genes in conjunction with the phylogenetic placement and quantification of the *nxrB* genotypes suggests complete ammonia-oxidizing (comammox) and nitrite-oxidizing *Nitrospira* populations co-exist with relatively equivalent and stable abundances in this system. It appears RAS biofilters harbor complex microbial communities whose composition can be affected directly by typical system operations while supporting multiple ammonia oxidation lifestyles within the nitrifying consortium.

## Introduction

The development of aquacultural technology allows societies to reduce dependency on capture fisheries and offset the effects of declining fish numbers (Barange et al., 2014). Aquaculture production now accounts for nearly 50% of fish produced for consumption, and estimates indicate a five-fold increase in production will be required in the next two decades to meet societal protein demands (FAO, 2014). However, expanding production will increase the environmental impact of aquaculture facilities and raises important concerns regarding the sustainability of aquaculture practices. Recirculating aquaculture systems (RAS) have been developed to overcome pollution concerns and stocking capacity limits of conventional terrestrial aquaculture facilities (Chen et al., 2006; Martins et al., 2010). RAS offer several advantages over traditional flow-through systems including: 90–99% reduced water consumption (Verdegem et al., 2006; Badiola et al., 2012), more efficient waste management (Piedrahita, 2003), and potential for implementation at locations that decrease distance to market (Martins et al., 2010). RAS components are similar to those used in wastewater treatment, including solids capture and removal of nitrogenous waste from excess animal waste and undigested feed. The advancement of RAS technology and advantages over flow-through systems has led to increasing RAS use, especially among countries that place high value on minimizing environmental impacts (Badiola et al., 2012) and in urban areas where space is limiting (Klinger and Naylor, 2012).

Nitrifying biofilters are a critical component of most RAS and an important determinant of operational success. These biofilters are also cited as the biggest hurdle for RAS start-up and the most difficult component to manage once the RAS is in operation (Badiola et al., 2012). RAS biofilters act to remove nitrogenous waste byproducts generated by fish protein catabolism and oxidation processes. Ammonia and nitrite are of most concern to freshwater aquaculturalists, with the toxic dose of both nitrogen species depending on pH and the aquatic organism being reared (Lewis and Morris, 1986; Randall and Tsui, 2002). In RAS process engineering, designers typically cite the principle nitrifying taxa as *Nitrosomonas* spp. (ammonia-oxidizers) and *Nitrobacter* spp. (nitrite-oxidizers) (Kuhn et al., 2010) and model system capacity from these organisms' physiologies (Timmons and Ebeling, 2013). It is now clear *Nitrosomonas* and *Nitrobacter* are typically absent or in low abundance in freshwater nitrifying biofilters (Hovanec and DeLong, 1996) while *Nitrospira* spp. are common (Hovanec et al., 1998). More recent studies of freshwater aquaculture biofilters have expanded the nitrifying taxa present in these systems to include ammonia-oxidizing archaea (AOA), a variety of *Nitrospira* spp., and *Nitrotoga* (Sauder et al., 2011; Bagchi et al., 2014; Hüpeden et al., 2016). Further studies are needed to understand whether other nitrifying consortia co-inhabit RAS biofilters with *Nitrosomonas* and *Nitrobacter* spp., or if diverse assemblages of nitrifying organisms are characteristic of high-functioning systems. A more refined understanding of RAS biofilter nitrifying consortia physiology would inform system design optimization and could alter parameters that are now considered design constraints.

The non-nitrifying component of RAS biofilter communities also impact biofilter function. Heterotrophic biofilm overgrowth can limit oxygen availability to the autotrophic nitrifying community resulting in reduced ammonia-oxidation rates (Okabe et al., 1995). Conversely, optimal heterotrophic biofilm formation protects the slower-growing autotrophs from biofilm shear stress and recycles autotrophic biomass (Kindaichi et al., 2004). Previous studies have suggested the diversity of non-nitrifying microorganisms in RAS biofilters could be large and sometimes contain opportunistic pathogens and other commercially detrimental organisms (Schreier et al., 2010). However, most of these studies used low-coverage characterization methods (e.g., DGGE, clone libraries) to describe the taxa present, so the extent of this diversity and similarity among systems is relatively unknown. Recently, the bacterial community of a set of seawater RAS biofilters run with different salinity and temperature combinations was characterized with massively parallel sequencing technology (Lee et al., 2016). This study provided the first deeper examination of a RAS biofilter microbial community, and revealed a highly diverse bacterial community that shifted in response to environmental conditions but more consistent nitrifying assemblage typically dominated by *Nitrospira*-classified microorganisms.

In this study, we aimed to deeply characterize the bacterial and archaeal community structure of a commercial-scale freshwater RAS raising *Perca flavescens* (Yellow perch) employing a fluidized sand biofilter that has been in operation for more than 15 years. We hypothesized that the biofilter sand biofilm community would exhibit temporal variability linked to environmental changes associated with the animal rearing process and a diverse nitrifying assemblage. To address these questions, we used massively parallel sequencing to characterize the bacterial and archaeal biofilter community across depth and time gradients. We also identified and phylogenetically classified nitrification marker genes for the ammonia monooxygenase alpha subunit (*amoA*; Rotthauwe et al., 1997; Pester et al., 2012; van Kessel et al., 2015) and nitrite oxidoreductase alpha (*nxrA*; Poly et al., 2008; Wertz et al., 2008) and beta (*nxrB*; Pester et al., 2014) subunits present in the biofilter, and then tracked their abundance with biofilter depth and over the course of a fish rearing cycle.

## Materials and methods

### UWM biofilter description

**Figure 1:**
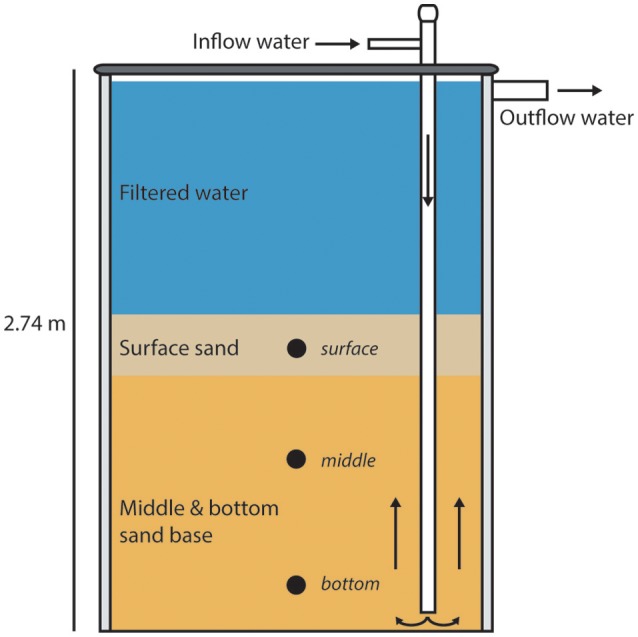
**llustration of the UW-Milwaukee recirculating aquaculture system (RAS) fluidized sand biofilter**. For illustration purposes only a single inflow pipe is shown. Nineteen of these pipes are present in the system. Water flow is depicted with directional arrows, sample locations are indicated by circles, and the biofilter height is listed.

All samples were collected from the University of Wisconsin-Milwaukee Great Lakes Aquaculture Facility RAS biofilter (UWM biofilter). Measured from the base, the biofilter stands ~2.74 m tall, with a diameter of ~1.83 m. The water level within the biofilter is ~2.64 m from the base, with the fluidized sand filter matrix extending to a height of ~1.73 m from the base. The biofilter is filled with Wedron 510 silica sand, which is fluidized to ~200% starting sand volume by the use of 19 schedule 40 PVC probes, each with a diameter of 3.175 cm. The probes receive influent from the solid waste clarifier, which upwells through the filter matrix. Samples for this study were taken at three depths within the fluidized sand biofilter, defined as surface (~1.32–1.42 m from biofilter base), middle (~0.81–0.91 m from biofilter base), and bottom (~0.15–0.30 m, from biofilter base). Depictions of the UWM biofilter and sample sites are shown in Figure 1. The maximum flow rate of the biofilter influent is 757 L per minute, which gives a hydraulic residence time of ~9.52 min. Typical system water quality parameters are as follows (mean ± standard deviation): pH 7.01 ± 0.09, oxidation-reduction potential 540 ± 50 (mV), water temperature 21.7 ± 0.9 (°C), and biofilter effluent dissolved oxygen (DO) 8.20 ± 0.18 mg/L. The biofilter is designed to operate maximally at 10 kg feed per day, which is based on the predicted ammonia production by fish protein catabolism at this feeding rate (Timmons and Ebeling, 2013).

### Sample collection, processing, and DNA extraction

Samples from the top of the biofilter matrix were collected in autoclaved 500 mL polypropylene bottles. Two samples from the surface of the biofilter were collected during the final 2 months of one Yellow perch rearing cycle and then immediately before the initiation of a new rearing cycle in the system. After stocking the system with fish, samples were collected approximately every week through the first half of the new rearing cycle (the strains of Yellow perch present during this study need ~9 months to grow to market size). Following collection, water from the biofilter matrix samples was decanted into a second sterile 500 mL bottle for further processing. Then, approximately 1 g wet weight sand was removed from the sample bottle and frozen at −80°C for storage prior to DNA extraction. Water samples were filtered onto 0.22 μm filters (47 mm mixed cellulose esters, EMD Millipore, Darmstadt, Germany), frozen at −80°C, and macerated with a sterilized spatula prior to DNA extraction. To separately address the spatial distribution of bacterial taxa, depth samples were taken from the filter matrix by using 50 mL syringes with attached weighted Tygon tubing (3.2 mm ID, 6.4 mm OD; Saint-Gobain S.A., La Défense, Courbevoie, France). Samples were binned into categories by approximate distance from the filter base as surface, middle and bottom. Tubing was sterilized with 10% bleach and rinsed 3X with sterile deionized water between sample collections. DNA was extracted separately from biofilter sand and water samples (~1 g wet weight and 100 mL, respectively) using the MP Bio FastDNA® SPIN Kit for Soil (MP Bio, Solon, OH, USA) according to the manufacturer's instructions except that each sample underwent 2 min of bead beating with the MP Bio FastDNA® SPIN kit's included beads at the Mini-BeadBeater-16's only operational speed (Biospec Products, Inc., Bartlesville, OK, USA). DNA quality and concentration was checked using a NanoDrop® Lite (Thermo Fisher Scientific Inc., Waltham, MA, USA). Sample details and associated environmental data and molecular analyses are listed in Table S1.

### Ammonia and nitrite measurements

For both the time series and depth profiles, a Seal Analytical AA3 Autoanalyzer (Seal Analytical Inc., Mequon, WI, USA) was used to quantify ammonia and nitrite, using the manufacturer's supplied phenol and sulfanilamide protocols on two separate channels. To quantify only nitrite, the cadmium reduction column was not incorporated into the Auto Analyzer. RAS operators recorded all other chemical parameters from submerged probes measuring temperature, pH, and oxidation-reduction potential. Per the laboratory standard operating procedure, RAS operators used Hach colorimetric kits to measure rearing tank concentrations of ammonia and nitrite.

### 16S rRNA gene sequencing

To maximize read depth for a temporal study of the biofilter surface communities, we used the illumina HiSeq platform and targeted the V6 region of the 16S rRNA gene for *Archaea* and *Bacteria* separately. In total, we obtained community data from 15 dates for the temporal analysis. To interrogate changes in the spatial distribution of taxa across depth in the biofilter and obtain increased taxonomic resolution, we used 16S rRNA gene V4-V5 region sequencing on an illumina MiSeq. We obtained samples from three depths *n* = 5 for the surface, *n* = 5 for the middle, and *n* = 4 for the bottom. Sample metadata are listed in Table S1. Extracted DNA samples were sent to the Josephine Bay Paul Center at the Marine Biological Laboratory (V6 *Archaea* and V6 *Bacteria*; V4-V5 samples from 12/8/2014 to 2/18/2015) and the Great Lakes Genomic Center (V4-V5 samples from 11/18/2014, 12/2/2014, 12/18/2014) for massively parallel 16S rRNA gene sequencing using previously published bacterial (Eren et al., 2013) and archaeal (Meyer et al., 2013) V6 illumina HiSeq and bacterial V4-V5 illumina MiSeq chemistries (Huse et al., 2014b; Nelson et al., 2014). Reaction conditions and primers for all illumina runs are detailed in the aforementioned citations, and may be accessed at: https://vamps.mbl.edu/resources/primers.php#illumina. Sequence run processing and quality control for the V6 dataset are described in Fisher et al. (2015), while CutAdapt was used to trim the V4-V5 data of low quality nucleotides (phred score <20) and primers (Martin, 2011; Fisher et al., 2015). Trimmed reads were merged using Illumina-Utils as described previously (Newton et al., 2015). Minimum entropy decomposition (MED) was implemented on each dataset to group sequences (MED nodes = operational taxonomic units, OTUs) for among sample community composition and diversity analysis (Eren et al., 2015). MED uses information uncertainty calculated via Shannon entropy at all nucleotide positions of an alignment to split sequences into sequence-similar groups (Eren et al., 2015). The sequence datasets were decomposed with the following minimum substantive abundance settings: bacterial V6, 377; archaeal V6, 123; bacterial V4-V5, 21. The minimum substantive threshold sets the abundance threshold for MED node (i.e., OTU) inclusion in the final dataset. Minimum substantive abundances were calculated by dividing the sum total number of 16S rRNA gene sequences per dataset by 50,000 as suggested in the MED best practices (sequence counts are listed in Table S2). The algorithm Global Alignment for Sequence Taxonomy (GAST) was used to assign taxonomy to sequence reads (Huse et al., 2008), and the website Visualization and Analysis of Microbial Population Structures (VAMPS; Huse et al., 2014a), was used for data visualization.

### Comammox *amoA* PCR

To target comammox *Nitrospira amoA* for PCR and subsequent cloning and sequencing, *amoA* nucleotide sequences from van Kessel et al. (2015) and Daims et al. (2015) were aligned using MUSCLE (Edgar, 2004). The alignment was imported into EMBOSS to generate an *amoA* consensus sequence (Rice et al., 2000). Primer sequences were identified from the consensus using Primer3Plus (Untergasser et al., 2012), and the candidates along with the methane monooxygenase subunit A (*pmoA*) primers suggested by van Kessel et al. (2015), were evaluated against the consensus sequence in SeqMan Pro (DNAStar), using MUSCLE (Edgar, 2004). The *pmoA* forward primer (Luesken et al., 2011) and candidate primer COM_amoA_1R (this study; Table 1) offered the best combination of read length and specificity, and subsequently were used to amplify *amoA* genes from our samples.

**Table 1:** **Primer sets used for endpoint PCR and qPCR**.

**Target organisms**	**Gene target**	**Assay type**	**Forward primer**	**Reverse primer**	**Primer conc. (nM)**	**Approximate product size (BP)**	**Thermocycler temperature programs**	**Citation**
Betaproteobacteria AOB	*amoA*	Endpoint PCR	1F 5′-GGG GHT TYT ACTGGT GGT-3′	2R 5′-CCC CTC KGS AAA GCCTTC TTC-3′	300	490	1 × 95°C 5:00 min; 30 × 95°C 0:30 min, 53°C 0:30 min, 72°C 0:30 min; 1 × 72°C 7:00 min	Rotthauwe et al., 1997; Christman et al., 2011
Gammaproteobacteria AOB	*amoA*	Endpoint PCR	3F 5′-GGT GAG TGG GYTAAC MG-3′	4R 5′-GCT AGC CACTTT CTG-3′	300	560	1 × 95°C 5:00 min; 30 × 95°C 0:30 min, 48°C 0:30 min, 72°C 0:30 min; 1 × 72°C 7:00 min	Christman et al., 2011
Ammonia-oxidizing Archaea	*amoA*	Endpoint PCR	19F 5′-ATG GTC TGG YTWAGA CG-3′	616R 5′-GCC ATC CAB CKRTAN GTC CA-3′	300	637	1 × 95°C 5:00 min; 30 × 95°C 0:30 min, 50°C 0:30 min, 72°C 0:30 min; 1 × 72°C 7:00 min	Tourna et al., 2008; Pester et al., 2012
Comammox *amoA*	*amoA*	Endpoint PCR	pmoA-189b-F 5′-GGN GAC TGG GACTTY TGG-3′	Com_amoA_1_R 5′-CGA GAT CATGGT GCT GTG AC-3′	300	520	1 × 95°C 10:00 min; 35 × 95°C 0:40 min, 56°C 0:40 min, 72°C 0:15 min; 1 × 72°C 7:00 min	Fwd (Luesken et al., 2011) & Rev This Study
*Nitrobacter* spp.	*nxrA*	Endpoint PCR	F1nxrA 5′-CAG ACC GAC GTG TGCGAA AG-3′	R2nxrA 5′-TCC ACA AGG AACGGA AGG TC-3′	300	322	1 × 94°C 5:00 min; 35 × 94°C 0:30 min, 55°C 0:45 min, 72°C 1:00 min; 1 × 72°C 10:00 min	Fwd (Poly et al., 2008) and Rev (Wertz et al., 2008)
Non-Nitrobacter NOB	*nxrB*	Endpoint PCR	nxrB169f 5′-TAC ATG TGGTGG AAC A-3′	nxrB638r 5′-CGG TTC TGGTCR ATC A-3′	300	485	1 × 95°C, 5:00 min; 35 × 95°C 0:40 min, 50°C 0:40 min, 72°C 1:30 min; 1 × 72°C 10:00 min	Pester et al., 2014
UWM AOA - Total	*amoA*	qPCR	Arch-amoAF 5′-CTG ACT GGG CGT GGACAT CA-3′	Arch-amoAR 5′-CCC AAT GCA AACCAT GCA CC-3′	200	170	1 × 95°C 2:00 min; 40 × 95°C 0:05 min, 62°C 0:45 min	This Study
UWM Nitroso - 1	*amoA*	qPCR	Beta-amoA-m1-F 5′-TCG AAC AAG GTT CACTCC GTA C-3′	Beta-amoA-m2-R 5′-ACA AAC GCT GAG AAGAAC GC-3′	200	70	1 × 95°C 2:00 min; 40 × 95°C 0:05 min, 61°C 0:45 min	This Study
UWM Nitroso - 2	*amoA*	qPCR	Beta-amoA-O2-F 5′-ATT TGG ACC GAC CCACTT ACC-3′	Beta-amoA-O2-R 5′-TAT GAC CAC CAA ACGTAC GC-3′	200	145	1 × 95°C 2:00 min; 40 × 95°C 0:05 min, 60°C 0:45 min	This Study
*Nitrospira nxrB* uwm-1	*nxrB*	qPCR	NitrospiraG1-a-F 5′-TAT GGG GTG TTC GAAGGG ATG-3′	NitrospiraG1-a-R 5′-ATG TTC ACG AAG CGCCAT TC-3′	200	104	1 × 95°C 2:00 min; 40 × 95°C 0:05 min, 67°C 0:45 min	This Study
*Nitrospira nxrB* uwm-2	*nxrB*	qPCR	NitrospiraG2-a-F 5′-ACG TCA AAA TCA CGCAGC TG-3′	NitrospiraG2-a-R 5′-CGG CAT CGA AAA TGGTCA TCC-3′	200	123	1 × 95°C 2:00 min; 40 × 95°C 0:05 min, 65°C 0:45 min	This Study
Comammox UWM *amoA*	*amoA*	qPCR	UWM_comammox_amoA_F1 5′-CGG ACT ACA TGGGCT TTG C-3′	UWM_comammox_amoA_R1 5′-GAG CCC ACT TCGATC ATC C-3′	200	70	1 × 95°C 2:00 min; 40 × 95°C 0:05 min, 59°C 0:45 min	This Study

### Clone library construction and phylogenetic analysis

Multiple endpoint PCR approaches were used to investigate the nitrifying community composition of the RAS fluidized sand biofilter for *amoA* (*Gammaproteobacteria, Betaproteobacteria, Archaea*, and comammox *Nitrospira*), *nxrA* (*Nitrobacter* spp.), and *nxrB* (non-*Nitrobacter* NOB). The primer sets and reaction conditions used are listed in Table 1. All endpoint PCR reactions were carried out at a volume of 25 μl: 12.5 μl 2x Qiagen PCR master mix (Qiagen, Hilden, Germany), 1.5 μl appropriate primer mix (F&R), 0.5 μl bovine serum albumin (BSA), 0.75 μl 50 mM MgCl_2_, and 1 μl DNA extract.

DNA samples of biofilter water and sand from four different rearing cycle time-points were used to construct clone libraries of archaeal *amoA* and *Nitrospira* sp. *nxrB*. One sample from the center of the sand biofilter was used to construct clone libraries for betaproteobacterial *amoA* and comammox *amoA*. The center biofilter sample was chosen as it produced well-defined amplicons suitable for cloning target *amoA* genes. All PCR reactions for clone libraries were constructed using a TOPO PCR 2.1 TA cloning kit plasmid (Invitrogen, Life Technologies, Carlsbad, CA). Libraries were sequenced on an ABI 3730 Sanger-Sequencer with M13 Forward primers. Vector plasmid sequence contamination was removed using DNAStar (Lasergene Software, Madison, WI).

Cloned sequences of *Betaproteobacteria amoA, Archaea amoA*, and *Nitrospira nxrB* from this study were added to ARB alignment databases from previous studies (Abell et al., 2012; Pester et al., 2012, 2014). Comammox *amoA* sequences from this study were aligned with those from van Kessel et al. (2015), Pinto et al. (2015), and Daims et al. (2015) using MUSCLE and imported into a new ARB database where the alignment was heuristically corrected before phylogenetic tree reconstruction. For the AOA, AOB, and *Nitrospira amoA* phylogenies, relationships were calculated using Maximum-Likelihood (ML) with RAxML on the Cipres Science Gateway (Miller et al., 2010; Stamatakis, 2014) and Bayesian inference (BI) using MrBayes with a significant posterior probability of <0.01 and the associated consensus tree (Abell et al., 2012; Pester et al., 2012, 2014) from ARB incorporated into a tree block within the input nexus file to reduce calculation time (Miller et al., 2010; Ronquist et al., 2012). Consensus trees were then calculated from the ML and BI reconstructions using ARB's consensus tree algorithm (Ludwig et al., 2004).

The *Nitrospira nxrB* sequences generated in this study were significantly shorter than those used for *nxrB* phylogenetic reconstruction in Pester et al. (2014), so we did not perform phylogenetic reconstructions as with the other marker genes. Instead, the UWM Biofilter and *Candidatus* Nitrospira nitrificans sequences were added to the majority consensus tree from Pester et al. (2014) using the Quick-Add Parsimony tool of the ARB package (Ludwig et al., 2004). This tool uses sequence similarity to add sequences to pre-existing trees without changing the tree topology.

### qPCR assays for target marker genes

Quantitative PCR assays were designed to differentiate two *Nitrospira nxrB* genotypes and two *Nitrosomonas amoA* genotypes in our system. Potential qPCR primer sequences were identified using Primer3Plus (Untergasser et al., 2012) on MUSCLE (Edgar, 2004) generated alignments in DNAStar (Lasergene Software, Madison, WI). Primer concentrations and annealing temperatures were optimized for specificity to each reaction target. Primers were checked using Primer-BLAST on NCBI to ensure the assays matched their target genes. The newly designed primers were tested for between genotype cross-reactivity using the non-target genotype sequence in both endpoint and real time PCR dilution series. After optimization, all assays amplified only the target genotype. Due to high sequence similarity between the two archaeal *amoA* genotypes (>90% identity) in our system, a single qPCR assay to target both genotypes was developed using the steps described above. The two closely related sequence types were pooled in equimolar amounts for reaction standards. A comammox *amoA* qPCR primer set was developed using the same methods as the other assays presented in this study. All assay conditions are listed in Table 1. All qPCR assays were run on an Applied Biosystems StepOne Plus thermocycler (Applied Biosystems, Foster City, CA). Cloned target genes were used to generate standard curves from 1.5 × 10^6^ to 15 copies per reaction. All reactions were carried out in triplicate, with melt curve and endpoint confirmation of assays (qPCR standard curve parameters and efficiency are listed in Table S3).

### Statistics and data analysis

Taxonomy-based data were visualized with heatmaps constructed in the R statistical language (R Core Team, 2014), by implementing functions from the libraries gplots, Heatplus from Bioconductor Lite, VEGAN, and RColorBrewer. MED nodes were used in all sample diversity metrics. The EnvFit function in the VEGAN (Oksanen et al., 2015) R package was used to test the relationship between RAS observational data and changes in the biofilter bacterial community composition. Pearson's correlations were calculated using the Hmisc package in R (Harrell, 2016) to test whether 16S rRNA, *amoA*, and *nxrB* gene copies correlated over time. Kruskal–Wallis rank sum tests were performed in the R base statistics package (R Core Team, 2014) to test whether the populations of the aforementioned genes were stratified by depth. The ADONIS function from VEGAN was used on the V4-V5 depth dataset to test the significance of the observed Bray-Curtis dissimilarity as a function of depth categorical factors, with strata = NULL since the same biofilter was sampled multiple times.

### Biomass model

To determine whether the observed ammonia removal could provide the energy needed to support the number of potential ammonia-oxidizing microorganisms (AOM) in the biofilter as quantified via qPCR, we modeled steady-state biomass concentration from measured ammonia oxidation with the following equation:

$$\begin{array}{l} X_{AO}=\frac{\theta x}{\theta }{\left[\frac{Yao}{1+{b_{AO}}^{*}\theta_{x}}\right]}^{*}\Delta S_{NH3} \end{array}$$

*X*_*AO*_ is defined as the biomass concentration of ammonia oxidizers in milligrams per liter in previous models (Mußmann et al., 2011), however, in this study we converted to cells per wet gram of sand by identifying the mean grams of sand per liter water in the biofilter. Θ_x_ is the mean cell residence time (MCRT) in days and was unknown for the system. Θ is the hydraulic retention time in days, which, is ~9.52 min, or 0.0066 days in this system. *Y*_*AO*_ is the growth yield of ammonia oxidizers, and *b*_*AO*_ is the endogenous respiration constant of ammonia oxidizers, which were estimated as 0.34 kg volatile suspended solids (VSS)/kg NH4^+^−N and 0.15 d^−1^ from Mußmann et al. (2011). Δ*S*_*NH*3_ is the change in substrate ammonia concentration between influent and effluent in mg/L. To calculate *X*_*AO*_, or biomass concentration, we used the mean cell diameter (0.96 μm) for *Candidatus* Nitrosocosmicus franklandus (Lehtovirta-Morley et al., 2016) to calculate the biovolume of a single cell, and used the conversion factor of 310 fg^*^C/μm^3^ (Mußmann et al., 2011) to relate biovolume to endogenous respiration. The modeled biomass concentration was plotted vs. a range of potential MCRT for a RAS fluidized sand filter (Summerfelt, Personal communication). The results of all *amoA* qPCR assays were combined to estimate total ammonia-oxidizing microorganism biomass in copy numbers per gram wet weight sand. Modeled biomass was then compared to our AOM qPCR assay results. A commented R-script for the model is available on GitHub (https://github.com/rbartelme/BFprojectCode.git).

### NCBI sequence accession numbers

Bacterial V6, V4-V5, and Archaeal V6 16S rRNA gene sequences generated in this study are available from the NCBI SRA (SRP076497; SRP076495; SRP076492). Partial gene sequences for *amoA* and *nxrB* are available through NCBI Genbank and have accession numbers KX024777–KX024822.

## Results

### Biofilter chemistry results

RAS operations data was examined from the beginning of a Yellow perch rearing cycle until ~6 months afterward. The mean biofilter influent concentrations of ammonia and nitrite were, respectively, 9.02 ± 4.76 and 1.69 ± 1.46 μM. Biofilter effluent ammonia concentrations (3.84 ± 7.32 μM) remained within the toxicological constraints (<60 μM) of *P. flavescens* reared in the system. On occasion, nitrite accumulated above the recommended threshold of 0.2 μM in both the rearing tank (0.43 ± 0.43 μM) and biofilter effluent (0.73 ± 0.49 μM). No major fish illnesses were reported during the RAS operational period. Environment and operations data are listed in Table S1.

### Bacterial and archaeal assemblages within the biofilter

The characterization of the RAS biofilter bacterial community revealed that both the sand-associated and water communities were diverse at a broad taxonomic level; 17 phyla averaged >0.1% in each of the biofilter sand and water bacterial communities (See Table S2 for sample taxonomic characterization to genus). *Proteobacteria* (on average, 40% of biofilter sand community sequences and 40% of water sequences) and *Bacteroidetes* (18% in sand, 33% in water) dominated both water and sand bacterial communities. At family-level taxonomic classification, the biofilter sand-associated community was distinct from the water community. The greatest proportion of sequences in the sand samples were classified to the bacterial groups, *Chitinophagaceae* (mean relative abundance, 12%), *Acidobacteria* family unknown (9%), *Rhizobiales* family unknown (6%), *Nocardioidaceae* (4%), *Spartobacteria* family unknown (4%), and *Xanthomonadales* family unknown (4%), while the water samples were dominated by sequences classified to *Chitinophagaceae* (14%), *Cytophagaceae* (8%), *Neisseriaceae* (8%), and *Flavobacteriaceae* (7%). At the genus-level *Kribbella, Chthoniobacter, Niabella*, and *Chitinophaga* were the most numerous classified taxa, each with on average >3% relative abundance in the biofilter samples.

Using Minimum Entropy Decomposition (MED) to obtain highly discriminatory sequence binning, we identified 1261 nodes (OTUs) across the bacterial dataset. A MED-based bacterial community composition comparison (Figure 1) supported the patterns observed using broader taxonomic classification indicating that the biofilter sand-associated community was distinct from the assemblage present in the biofilter water.

In contrast to the large diversity in the bacterial community, we found the archaeal community to be dominated by a single taxonomic group, affiliated with the genus *Nitrososphaera*

This taxon made up >99.9% of the *Archaea*-classified sequences identified in the biofilter samples (Table S2). This taxon also was represented almost completely by a single sequence (>95% of *Archaea*-classified sequences) that was identical to a number of database deposited *Thaumarchaeota* sequences, including the complete genome of *Candidatus* Nitrosocosmicus oleophilus (CP012850), along with clones from activated sludge, wastewater treatment, and freshwater aquaria (KR233006, KP027212, KJ810532–KJ810533).

**Figure 2:**
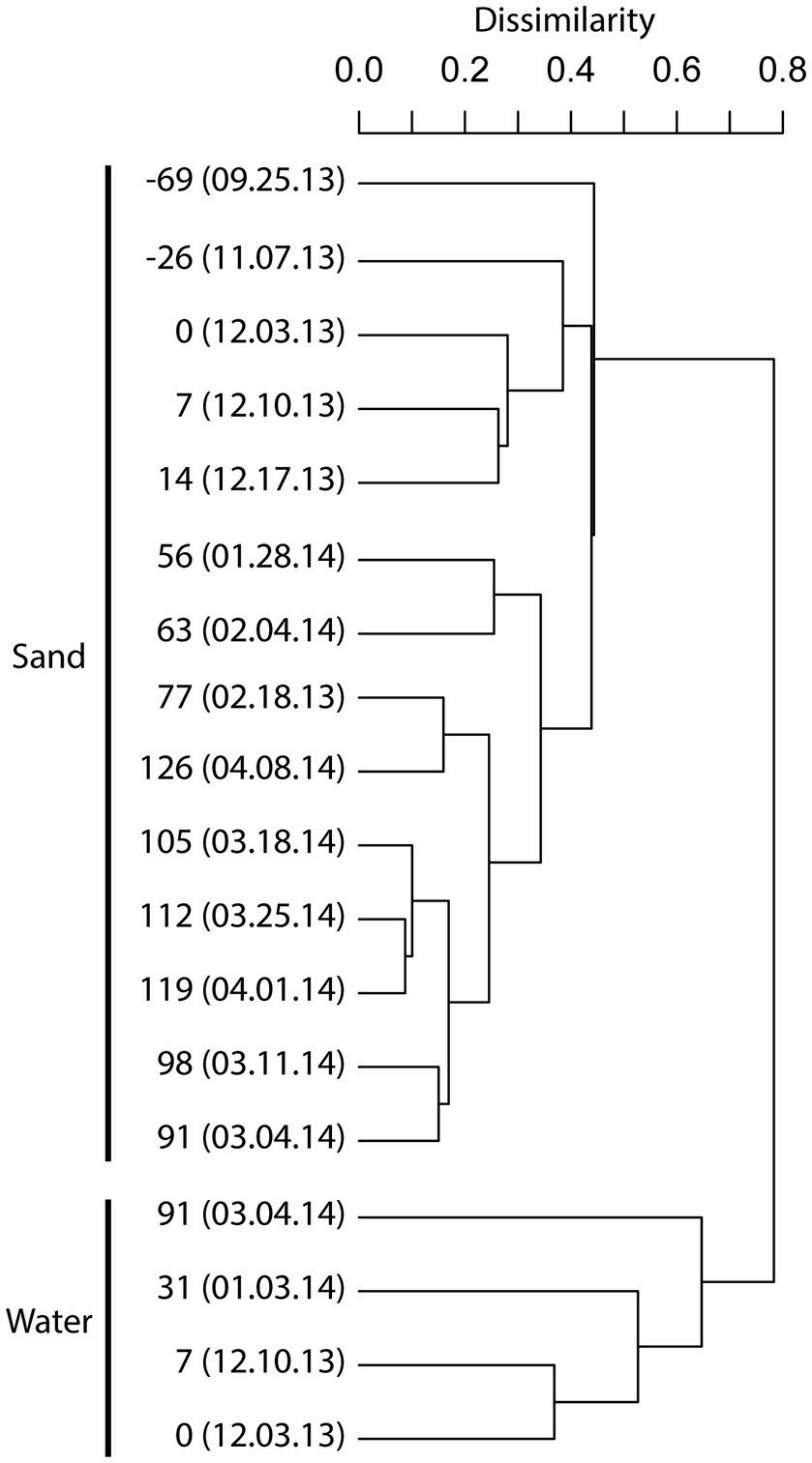
**Dendrogram illustrating the bacterial community composition relationships among biofilter sand and biofilter water samples**. A complete-linkage dendrogram is depicted from Bray–Curtis sample dissimilarity relationships based on Minimum Entropy Decomposition node distributions among samples (V6 dataset). The leaves of the dendrogram are labeled with the day count, where 0 represents the beginning of a fish rearing cycle. Negative numbers are days prior to a new rearing cycle. The day count is followed by the date sampled (mm.dd.yy). See Table S1 for sample metadata.

**Figure 3:**
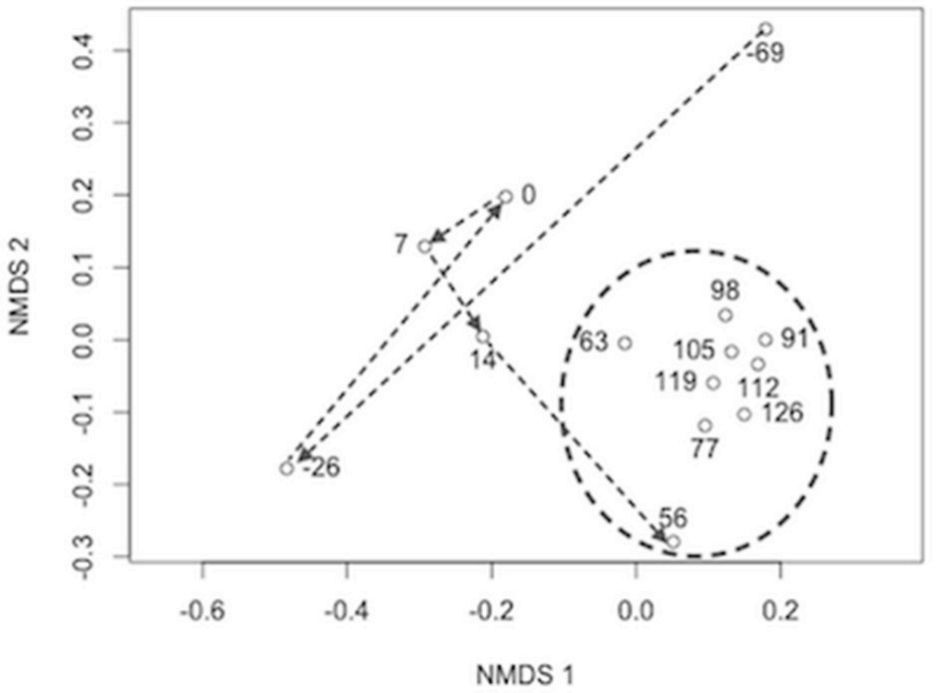
**Non-metric multidimensional scaling plot of Bray–Curtis bacterial community composition dissimilarity between sample time points**. nMDS Stress = 0.07 and dimensions (k) = 2. Arrows indicate the sample progression through time from the end of one rearing cycle (daynumber −69 and −26), to a period with no fish (0), and into the subsequent rearing cycle (7–126). The circle indicates samples taken after fish had grown to a size where feed type and amount were stabilized (3 mm pelleted feed diet and 3–7 kg of feed per day).

The initial biofilter community composition characterization revealed distinct communities between the biofilter sand and decanted biofilter water (Figure 2). Based on this data and that fluidized-bed biofilter nitrification occurs primarily in particle-attached biofilms (Schreier et al., 2010), we focused our further analyses on the biofilter sand matrix. In the sand samples, we observed a significant change in bacterial community composition (MED nodes) over time (Table 2). The early portion of the study, which included a period while market sized Yellow perch were present in the system (sample −69 and −26), a fallow period following fish removal (sample 0), and time following re-stocking of mixed-age juvenile fish (sample 7 and 14), had a more variable bacterial community composition (Bray-Curtis mean similarity 65.2 ± 6.5%) than the remaining samples (*n* = 9) collected at time points after an adult feed source had been started (20.0 ± 6.4%, Figure 3). Several operational and measured physical and chemical parameters, including oxidation-reduction potential, feed size, conductivity, and biofilter effluent nitrite were correlated (*p* < 0.05) with the time-dependent changes in bacterial community composition (see Table 2 for environmental correlation results).

**Table 2:** **Environmental variable to bacterial community composition correlations**.

**Variable^a^^,^^b^**	**Dim1**	**Dim2**	***R*^2^**	**Pr(>r)**
Days From Start	0.836	0.548	0.94	0.002
Number of Fish	−0.839	−0.544	0.77	0.024
Fish Mortalities	0	0	0	1
Culled Fish	0	0	0	1
System pH	−0.454	0.891	0.03	0.911
Air Temperature	0.844	0.537	0.39	0.326
Water Temperature	0.752	0.659	0.69	0.05
Conductivity	0.970	−0.242	0.82	0.042
System Ammonia	0.651	0.759	0.50	0.19
System Nitrite	0.823	−0.568	0.87	0.011
Biofilter PSI	0.473	0.881	0.70	0.081
Biofilter Influent Ammonia	0.297	0.955	0.63	0.097
Biofilter Effluent Ammonia	−0.582	0.813	0.03	0.949
Biofilter Influent Nitrite	0.687	0.727	0.69	0.057
Biofilter Effluent Nitrite	0.782	0.623	0.81	0.01
ORP	0.928	−0.374	0.82	0.021
Feed Size	0.991	−0.133	0.88	0.042
kg feed	0.798	0.603	0.47	0.19
Percent Variance Explained^c^	23.8	11.0	–	–

a *The V6 16S rRNA gene biofilter sand bacterial community composition data were related to the system metadata in Table S1 using environmental vector fitting of a principal coordinates analysis (Oksanen et al., 2015; VEGAN EnvFit function)*.

b *Days From Start, Days following the start of a rearing cycle; Culled fish, the number of fish removed from the system up to the point of sampling; System pH, pH in the rearing tank; ORP, oxidation reduction potential; Biofilter PSI is the pressure within the biofilter manifold, in pounds per square inch*.

c *Percent variance explained by the first and second axes in the bacterial community composition ordination*.

**Figure 4:**
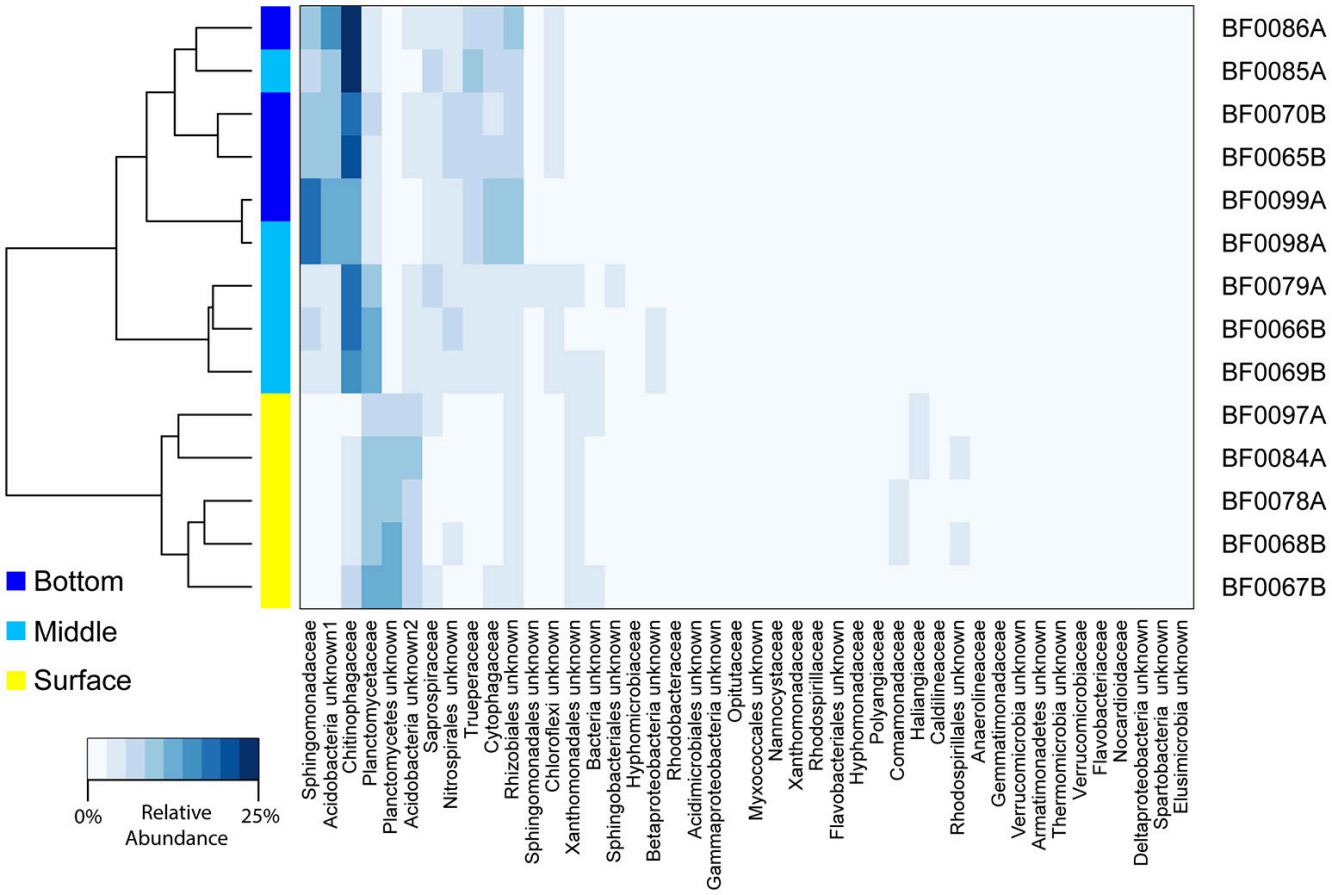
**Depth comparison of bacterial biofilter community composition**. A heatmap is depicted for all bacterial families with ≥1% relative abundance in any sample. Taxon relative abundance was generated from V4–V5 16S rRNA gene sequencing and is indicated with a scale from 0 to 25%. The dendrogram represents Bray-Curtis dissimilarity between sample community composition. Sample IDs are listed and sample depth is indicated by on the plot next to the dendrogram. Sample names correspond to sample metadata in Table S1.

Using a second sequence dataset (V4-V5 16S rRNA gene sequences), we examined the bacterial community composition associated with sand across a depth gradient (surface, middle, bottom). We found the bacterial communities in the top sand samples were distinct from those in the middle and bottom (ADONIS *R*^2^ = 0.74, *p* = 0.001; Figure 4). The *Planctomycetes* were a larger portion of the community in the surface sand (on average 15.6% of surface sand vs. 9.6% of middle/bottom sand), whereas the middle and bottom layers harbored a greater proportion of *Chitinophagaceae* (7.4% in surface vs. 16.8% in middle/bottom) and *Sphingomonadaceae* (2.4% in surface vs. 7.9% in middle/bottom; Figure 4).

### Nitrifying community composition and phylogeny

The massively parallel 16S rRNA gene sequencing data indicated bacterial taxa not associated with nitrification comprised the majority (~92%) of the sand biofilter bacterial community. In contrast, >99.9% of the archaeal 16S rRNA gene sequences were classified to a single taxon associated with known AOA. Among the bacterial taxa, *Nitrosomonas* represented <1% of the total community across all samples and no *Nitrobacter* sequences were obtained. We also were unable to amplify *Nitrobacter nxrA* genes (Figure S1) with a commonly used primer set (Poly et al., 2008; Wertz et al., 2008). In contrast, *Nitrospira* was fairly abundant, comprising 2–5% of the total bacterial community (Table S2).

**Figure 5:**
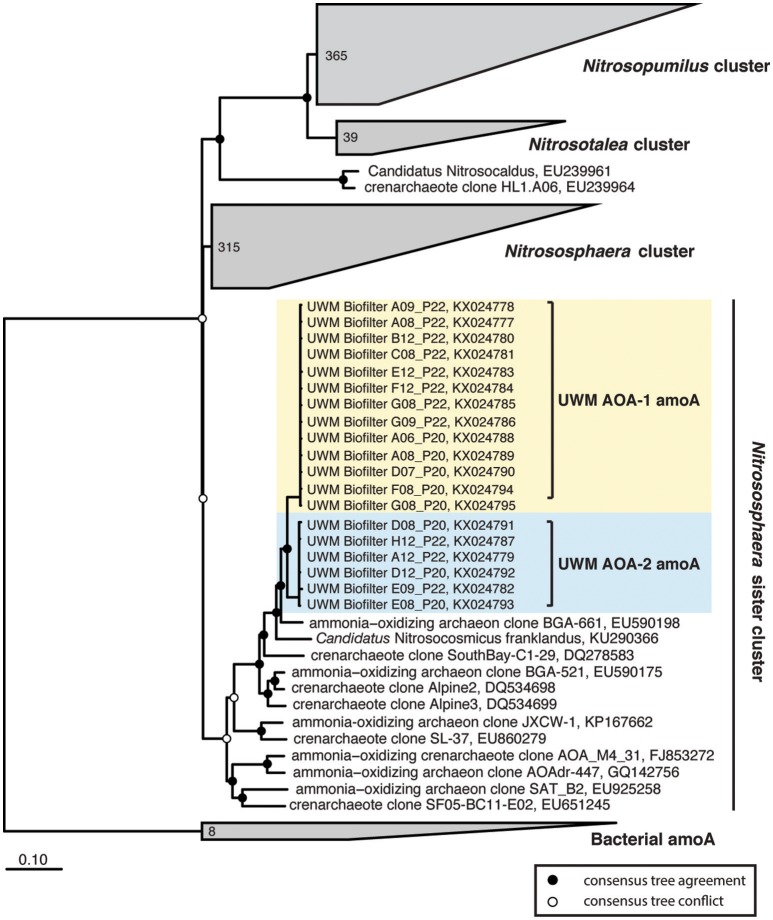
**Ammonia-oxidizing Archaea consensus tree**. A consensus phylogenetic tree was generated from maximum likelihood and Bayesian inference phylogenetic reconstructions. Consensus tree support is indicated by colored circles at tree nodes. Collapsed nodes and assigned names are based off of Pester et al. (2012). Clone and taxonomic names are followed by NCBI accession numbers. Ammonia-oxidizing archaea amoA sequences generated in this study are highlighted.

**Figure 6:**
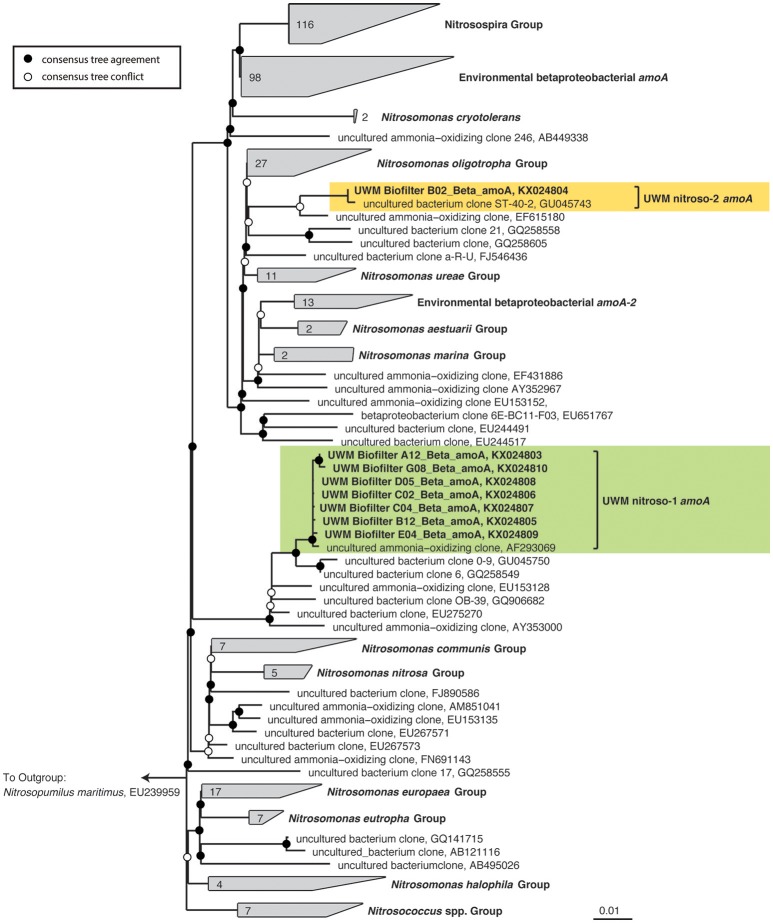
**Ammonia-oxidizing Bacteria consensus tree**. A consensus phylogenetic tree was generated from maximum likelihood and Bayesian inference phylogenetic reconstructions. Consensus tree support is indicated by colored circles at tree nodes. Collapsed nodes and assigned names are based off of Abell et al. (2012). Clone and taxonomic names are followed by NCBI accession numbers. The clade containing *Nitrosomonas amoA* genotype, UWM *nitroso*-1 *amoA* is highlighted in green, and UWM *nitroso*-2 *amoA* is highlighted in yellow.

In addition to the 16S rRNA gene community data, we amplified, cloned, and sequenced nitrifying marker genes representing the dominant nitrifying taxa in the UWM biofilter. The archaeal *amoA* sequences (KX024777–KX024795) clustered into two distinct genotypes, with an average nucleotide identity ranging from 97 to 99%. Both genotypes placed phylogenetically in the *Nitrososphaera* sister cluster (Figure 5), which includes the candidate genus, *Nitrosocosmicus* (Lehtovirta-Morley et al., 2016), but the sequences were most closely related to the *amoA* genes from Archaeon G61 (97% nucleotide identity; KR233005). Sequenced amplicons for betaproteobacterial *amoA* (KX024803–KX024810) also revealed the presence of two AOB genotypes affiliated with *Nitrosomonas*. These *Nitrosomonas* genotypes were most closely related (99% identity) to environmental sequences obtained from freshwater aquaria and activated sludge (Figure 6).

**Figure 7:**
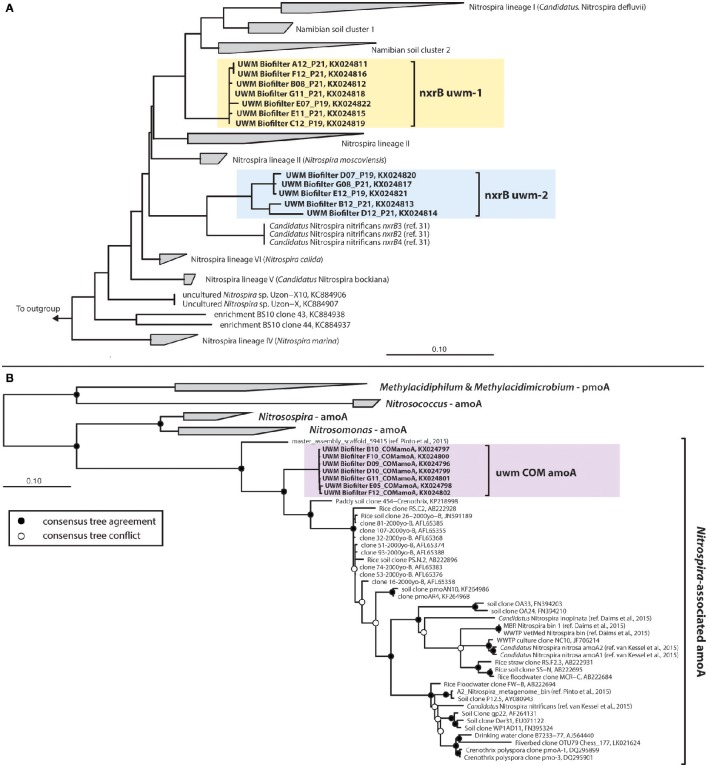
**Consensus phylogenetic trees for *Nitrospira*-like (A)** *nxrB* and **(B)** *amoA* genes. For the nxrB phylogeny, the consensus tree from Pester et al. (2014) is illustrated. The UWM Biofilter and *Candidatus* Nitrospira nitrificans sequences were added to this phylogenetic reconstruction with the Quick-Add Parsimony tool of the ARB package (Ludwig et al., 2004), so as not to change the tree topology. For the *amoA* phylogeny, a consensus phylogenetic tree was generated from maximum likelihood and Bayesian inference phylogenetic reconstructions. Consensus tree support is indicated by colored circles at tree nodes. Clone names are followed by NCBI accession numbers or a manuscript citation. In both trees, sequences generated in this study are highlighted with colored boxes.

The UWM biofilter sand also harbored two phylogenetically distinct and divergent clades of *nxrB* sequences (85–86% nucleotide identity between genotypes; KX024811–KX024822) affiliated with the genus *Nitrospira*. *Nitrospira nxrB* uwm-1 formed a clade distinct from cultivated *Nitrospira* spp. (~92% nucleotide identity to *Nitrospira bockiana*). *Nitrospira nxrB* uwm-2 clustered phylogenetically with *Nitrospira* spp., which have been implicated in complete nitrification (i.e., comammox; Daims et al., 2015; van Kessel et al., 2015; Figure 7A). Because of the association of *Nitrospira nxrB* uwm–2 with comammox *nxrB* sequences, we further examined the biofilter for the presence of *Nitrospira*-like *amoA* genes. We subsequently amplified a single *Nitrospira*-like *amoA* out of the biofilter samples, and phylogenetic inference placed this *amoA* on a monophyletic branch with currently known *Nitrospira amoA* sequences, but in a distinct cluster (Figure 7B) with a drinking water metagenome contig (Pinto et al., 2015) and a “Crenothrix *pmoA/amoA*” Paddy Soil Clone (KP218998; van Kessel et al., 2016). A link to ARB databases containing these data may be found at https://github.com/rbartelme/ARB_dbs.

### Temporal and spatial quantification of nitrification marker genes

**Figure 8:**
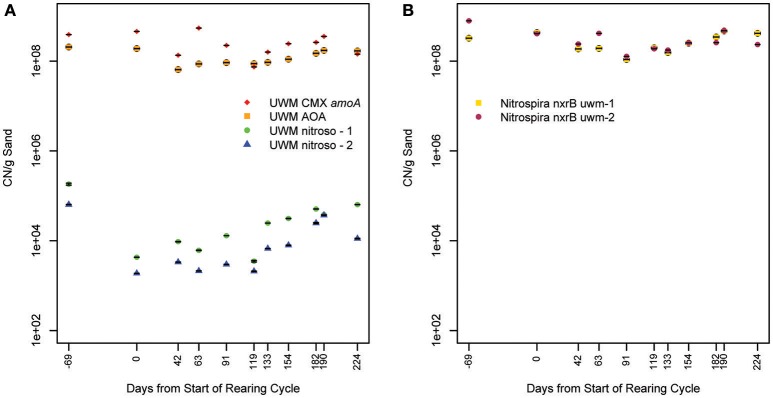
**Nitrification marker gene concentration over time**. Plot **(A)** illustrates *amoA* copy number (CN) per gram of biofilter sand and plot **(B)** *nxrB* CN per gram of biofilter sand for all identified genotypes. Standard deviation of triplicate qPCR reactions is indicated for each sample. The x-axis indicates time, with timepoint 0 representing the beginning of one fish rearing cycle. Samples collected in the previous rearing cycle are labeled with negative values. See Table S1 for sample metadata.

We investigated the temporal and spatial stability of the nitrifying organisms in the UWM biofilter by developing qPCR assays specific to identified *amoA* and *nxrB* genes. Within the ammonia-oxidizing community, the AOA and comammox-*Nitrospira* (*amoA* assay) had space-time abundance patterns distinct from that of the *Nitrosomonas* genotypes. For example, the AOA and comammox-*Nitrospira* were numerically dominant (range = 450–6500:1) to *Nitrosomonas* (combined UWM nitroso-1 and nitroso-2 genotypes) across all samples (Figure 8; Table 3). The AOA and comammox-*Nitrospira* also had more stable abundances over time [Coefficient of variation (CV) = 0.38 and 0.55 vs. 1.33 and 1.32 for nitroso-1 and nitroso-2; Figure 8], copy number concentrations that were less impacted by biofilter depth (Table 3), and comammox-*Nitrospira* were approximately 1.9x more abundant than AOA throughout the biofilter. Lastly, the two *Nitrosomonas amoA* genotypes exhibited a strong temporal abundance correlation (Pearson's *R* = 0.90, pseudo *p* = 0.0002) that was not shared with AOA or the comammox-*Nitrospira* (Pearson's *R* = 0.65 and 0.69, and pseudo *p* = 0.031 and 0.019, respectively).

**Table 3:** **Nitrification marker gene concentrations in biofilter sand**.

**qPCR Assay^a^**	**Bottom (CN/g)^b^**	**Middle (CN/g)**	**Surface (CN/g)**	**Significance^d^**
UWM AOA-Total (*amoA*)^c^	2.1 × 10^8^ ± 0.2 × 10^8^	2.6 × 10^8^ ± 0.8 × 10^8^	1.0 × 10^8^ ± 0.06 × 10^8^	χ^2^ = 5.4 and *p* = 0.07
UWM *Nitroso*–1 (*amoA*)	4.6 × 10^5^ ± 0.3 × 10^5^	3.6 × 10^4^ ± 1.3 × 10^4^	4.5 × 10^4^ ± 2.9 × 10^4^	χ^2^ = 5.6 and *p* = 0.06
UWM *Nitroso*–2 (*amoA*)	2.0 × 10^4^ ± 0.4 × 10^4^	4.0 × 10^3^ ± 1.7 × 10^3^	3.5 × 10^3^ ± 1.9 × 10^3^	χ^2^ = 5.4 and *p* = 0.07
*Nitrospira nxrB* uwm-1	5.8 × 10^8^ ± 1.0 × 10^8^	7.4 × 10^8^ ± 3.9 × 10^8^	4.6 × 10^8^ ± 1.3 × 10^8^	χ^2^ = 2.3 and *p* = 0.32
*Nitrospira nxrB* uwm-2	4.9 × 10^8^ ± 1.8 × 10^8^	4.6 × 10^8^ ± 2.1 × 10^8^	4.2 × 10^8^ ± 1.4 × 10^8^	χ^2^ = 0.35 and *p* = 0.84
*Comammox* (*amoA*)	3.5 × 10^8^ ± 0.7 × 10^8^	3.9 × 10^8^ ± 1.0 × 10^8^	2.5 × 10^8^ ± 0.9 × 10^8^	χ^2^ = 1.7 and *p* = 0.43

a *Mean and standard deviation are listed*.

b *Bottom, middle, and surface depth categories are defined as: surface (~1.32–1.42 m from biofilter base), middle (~0.81–0.91 m from biofilter base), and bottom (~0.15–0.30 m, from biofilter base)*.

c *For nxrB, n = 4, and for amoA n = 3. Corresponding samples are listed in Table S1*.

d *χ^2^ and P-values from Kruskal–Wallis Rank Sum assessment of depth as a significant factor in nitrification marker gene distribution*.

**Figure 9:**
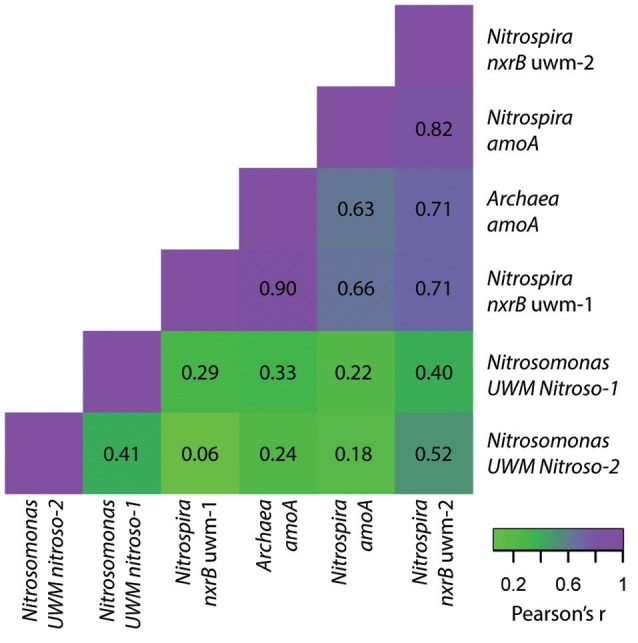
**Heatmap of abundance pattern correlations for nitrifier genotypes**. Pearson's correlation coefficient values (r) are listed and colored according to the strength of the abundance correlation between marker genes for each genotype. Purple colors indicate stronger correlations and green colors indicate weaker correlations.

Within the nitrite-oxidizing community, the abundance of both *Nitrospira* genotypes (*nxrB* uwm-1 and uwm-2) was in the range of 10^8^ CN/g sand, and each exhibited temporal and spatial (depth) abundance stability (Table 3; Figure 8). The two genotypes also exhibited abundance co-variance across all samples (Pearson's *R* = 0.71, pseudo *p* = 0.0002). Despite these abundance pattern similarities, the two genotypes had differential associations with other nitrifying taxa marker genes. Genotype uwm-1, which is phylogenetically associated with strict nitrite-oxidizers, had strong abundance co-variation with the AOA *amoA* (Pearson's *R* = 0.90, pseudo *p* ≤ 0.0001), while genotype uwm-2 (phylogenetically associated with comammox-*Nitrospira*) had a stronger relationship to the *Nitrospira amoA* (Pearson's *R* = 0.82, pseudo *p* ≤ 0.0001; Figure 9).

### Ammonia-oxidizing microorganism biomass model

**Figure 10:**
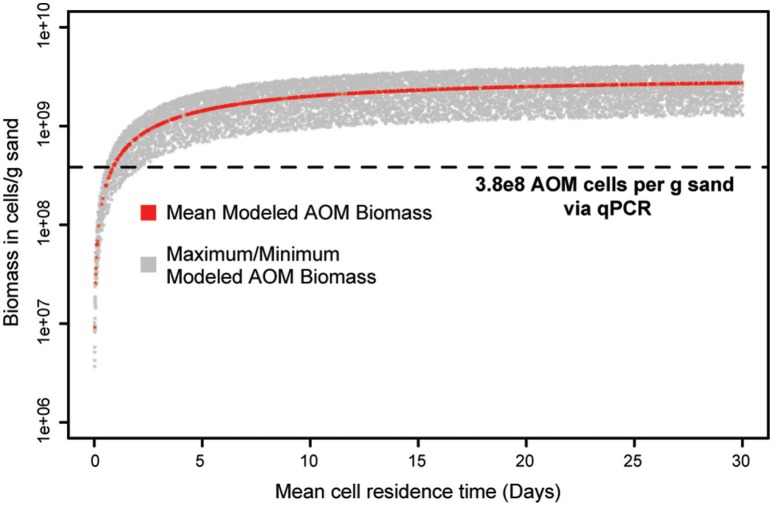
**Model output of ammonia-oxidizer cell concentration as a function of biofilter mean cell residence time (MCRT)**. The red line indicates ammonia-oxidizer cell abundance estimates from the mean change in ammonia concentration across the filter matrix as a function of mean cell residence time. The shaded gray region represents the range of cell abundance estimates from the minimum and maximum observed ammonia removal rates. The horizontal dashed line indicates qPCR estimated total ammonia-oxidizer abundance (ammonia-oxidizing *Archaea* + ammonia-oxidizing *Bacteria* + comammox *Nitrospira*) in the system.

The estimated cell densities for ammonia oxidizers in the biofilter were modeled as a function of mean cell residence time (MCRT). Since the biofilter MCRT was unknown, a range of values (1–30 days) was used in the model. The model suggests the combined estimated ammonia oxidizer cell densities (*Nitrosomonas* + AOA + commamox-*Nitrospira*) could be supported by the ammonia oxidation observed, and in fact over-estimated these densities. For example, the model indicates ammonia oxidizer biomass reaches near maximum by a mean cell residence time (MCRT) of 20 days (Figure 10). At this 20-day MCRT, the model indicates the ammonia removal rate measured could support ~6.2X more cells than we observed (Figure 10).

## Discussion

### Biofilter microbial community composition

In this study, we generated data that deeply explored the microbial community composition for a production-scale freshwater RAS nitrifying biofilter, expanding our understanding of the complexity of these systems beyond previous reports (Sugita et al., 2005; Sauder et al., 2011; Blancheton et al., 2013). This deeper coverage gave us the power to examine temporal and depth distributions for both total bacterial and archaeal communities and the potential nitrifying member consortia therein. In previous studies of freshwater RAS biofilters, *Actinobacteria, Gammaproteobacteria, Plantomycetes*, and *Sphingobacteria* were identified as dominant taxa, while at more refined taxonomic levels *Acinetobacteria, Cetobacterium, Comamonas, Flectobacillus, Flavobacterium*, and *Hyphomicrobium* were common (Sugita et al., 2005). All of these genera were present and relatively abundant (>0.5% total community; genus level taxonomic breakdown in Table S2) in our biofilter sand samples, suggesting there may be selection pressures for heterotrophs that act universally across systems. Some researchers have hypothesized that each RAS biofilter should have a unique microbial community composition shaped by operational controls and components implemented in the RAS (Sugita et al., 2005; Blancheton et al., 2013). In support of this idea, many of the most abundant bacterial genera in our system (e.g., *Kribbella, Niabella, Chitinophaga, Byssovorax, Hyphomicrobium*) had not been reported as abundant in other systems. While it is likely true that each microbial community assemblage will be unique among RAS biofilters, i.e., each biofilter has a unique “microbial fingerprint,” the low number of RAS biofilters with community composition information to date and the low sequencing depth within existing studies, prohibits making robust comparisons across systems and identifying underlying community composition trends that relate to system operations.

Different components of RAS are expected to have unique environmental selective pressures, and thus multiple distinct microbial communities should be present within a single RAS. Our community data indicates there are consistent and significant differences in the biofilter sand and water communities. These differences included community members that were ubiquitous in, but nearly exclusive to the water samples. These taxa could be remnant members derived from previous components in the system (e.g., rearing tank, clarifier), but the high shear force in a fluidized sand bed may make for inconsistent passage of these inflow microorganisms. The water samples also had decreased representation of prominent sand-associated taxa, including most known nitrifiers, so studies sampling biofilter outflow water would not represent accurately the microbial assemblages associated with nitrification. These observations support previous observations to the same effect, further lending support to the idea that a transient planktonic microbial assemblage is constantly moving through RAS components while an independent community develops on the biofilter media (Blancheton et al., 2013).

Our time series indicates RAS biofilter bacterial community composition change correlates with environmental parameter shifts related to fish growth (i.e., number of fish, water temperature, conductivity, oxidation-reduction potential, and feed size). This result is consistent with the hypothesis that biofilter bacterial community variation follows feed and fish growth driven shifts in the C/N ratio (Michaud et al., 2006, 2014). The community variability is seemingly confined to the non-nitrifying members of the biofilter, as the dominant nitrifying organisms changed little in composition or abundance over time. Sampling different depths in the biofilter revealed distinct microbial communities in each sand stratum, suggesting a potential partitioning across physical and chemical gradients within the biofilter. In contrast to the observed temporal variation, these differences were present both in the heterotrophic assemblages, and in the abundance of nitrifiers. It appears this biofilter maintains a stable, but depth partitioned nitrifying community in the midst of a shifting bacterial community, whose composition is linked to variation in nutrient inputs, ultimately stemming from the output of fish growth.

Generally, the RAS biofilter heterotrophic microbial community is viewed only as competing with nitrifiers for resources, and system design guidelines recommend operations based on this premise (Okabe et al., 1995). However, this view may confine further development of biofilter technology, as it is becoming apparent that the heterotrophic community context can play a broader role in nitrification. Our data clearly indicates the heterotroph community varies substantially during “typical” fish rearing cycles. It is possible under some scenarios that these changes could impact nitrification. For example, certain heterotrophs are known to enhance nitrification rates in *Nitrosomonas* and *Nitrobacter* bioreactors (Sedlacek et al., 2016). It is unknown whether these interactions extend to other ammonia and nitrite-oxidizing taxa or other systems, but the interplay between heterotrophs and nitrifiers as a means to enhance nitrification rates in RAS should be investigated. Further data across systems and over longer periods in a single system are also needed to bound “normal” vs. stochastic system variability and identify key taxa or community assembly principles governing RAS.

### Nitrifying consortia

Prior to metagenomic studies, members of a few bacterial clades were believed to be responsible for ammonia oxidation. The isolation of the first ammonia-oxidizing archaeon, *Nitrosopumilus maritimus*, altered global nitrification models (Könneke et al., 2005). AOA are ubiquitous in both natural and engineered environments and are seemingly differentiated by niche from ammonia-oxidizing bacteria (AOB) based on ammonia concentration, where AOA outcompete AOB at relatively low concentrations (Hatzenpichler, 2012). This relationship appears to extend to freshwater biofilters, as it was shown recently that AOA dominate in freshwater aquaria biofilters when ammonia concentrations are low (<30 μM; Pester et al., 2011). Our data support these previous findings, as AOA were 6 × 10^5^ times more abundant than both *Nitrosomonas* genotypes in the UWM biofilter, which maintains similarly low influent ammonia concentrations (mean = 9 μM). AOA showed little abundance variation with depth or over time (<3X change) while *Nitrosomonas* exhibited an order of magnitude greater abundance during later periods in the fish rearing cycle and deeper in the biofilter (Table 3). System ammonia is highest late in the rearing cycle (Table S1) and presumably deeper in the biofilter, which is nearest to the influent ports.

Although, AOA were numerically dominant over AOB, a presumed third ammonia-oxidizer was also present in the biofilter sand matrix. Identification of *Nitrospira*-like *amoA* (Figure 7B) in the biofilter and the strong correlation between the abundance of the *Nitrospira nxrB uwm-2* gene and this *Nitrospira amoA*, suggests a complete ammonia-oxidizing *Nitrospira* spp. resides in the UWM biofilter. In fact, we found that the comammox *amoA* was the most abundant ammonia-oxidizing gene in the biofilter (on average 1.9X that of AOA *amoA*). Similar to the AOA, the comammox *Nitrospira* exhibited little abundance variation with depth or over time, which suggests the AOA and comammox *Nitrospira* stably co-exist throughout this system. The comammox reaction is predicted to be competitive in systems with limited substrate influx, and comammox *Nitrospira* have proven to be common in drinking water systems (Pinto et al., 2015). Part of the initial discovery of comammox included a comammox *Nitrospira* from a RAS (van Kessel et al., 2015), but in the anoxic portion of a trickling biofilter. Thus, RAS biofilters, which often have a municipal water source and relatively low nutrient influx may be a common reservoir of comammox *Nitrospira* colonization.

The physiology of the UWM RAS biofilter AOA cannot be interpreted from our dataset, but both the AOA genotypes cluster phylogenetically within the *Nitrososphaera* sister cluster, which is represented mainly by cloned *amoA* sequences from soil, sediment, and some AOA associated with freshwater aquaria. Recently an organism given the name *Candidatus* Nitrosocosmicus franklandus (Lehtovirta-Morley et al., 2016) was isolated from the *Nitrososphaera* sister cluster. *Ca. Nitrosocosmicus* spp. appear to be suited to tolerate higher concentrations of ammonia and nitrite than other AOA, and are capable of ureolytic growth (Lehtovirta-Morley et al., 2016), both of which could be beneficial traits in RAS environments. AOA, now have been detected in freshwater, brackish, and saline RAS that also span a variety of cultured species, ranging from finfish to crustaceans (Urakawa et al., 2008; Sauder et al., 2011; Sakami et al., 2012). Given the common AOA dominance over *Nitrosomonas* in RAS nitrifying biofilters, including in our study system, a greater understanding of AOA ecophysiology is needed to understand how system designs could be used to maximize AOA capabilities.

Although AOA appear widespread in RAS biofilters, the presence of AOA with comammox *Nitrospira* in our system suggests understanding AOA physiology may be only a part of understanding RAS biofilter nitrification. It is clear this environment generally favors the proliferation of organisms thought to be high affinity, low substrate specialists and can support a complex nitrifying consortium. However, further work is needed to understand how ammonia-oxidation partitions between the various ammonia-oxidizers competing for substrate and how system operations can take advantage of potentially flexible ammonia-oxidizer physiologies.

In our system, we did not detect *Nitrobacter*, whose physiological constraints are often used when calculating RAS biofiltration capacity. Instead we identified *Nitrospira* as the dominant nitrite-oxidizing bacteria (NOB). *Nitrospira* are generally considered *K*-strategist NOB favoring oligotrophic environments, while *Nitrobacter* are *r*-strategist copiotrophs (Nowka et al., 2015). *Nitrospira* uwm-1 exhibited a strong abundance pattern correlation with AOA, had abundances roughly equal (~10^8^ *nxrB* CN/g sand) to that of the AOA, and clustered phylogenetically with known nitrite-oxidizing *Nitrospira*. Together, this suggests *Nitrospira* uwm-1 is the primary strict nitrite-oxidizing bacterium in this biofilter. The dominance of *Nitrospira* in this system and several other RAS (Schreier et al., 2010; van Kessel et al., 2010; Auffret et al., 2013; Brown et al., 2013; Kruse et al., 2013) indicates there is a versatile metabolic network driving RAS biofilter nitrification. For example, nitrite-oxidizing *Nitrospira* spp. possess a diverse array of metabolic pathways, and have been shown experimentally to hydrolyze urea and cyanate to ammonia, thereby initiating nitrification through cross-feeding with AOA/AOB. This process is counter to the supposed role of nitrite oxidizers solely as converters of nitrite to nitrate (Daims et al., 2016). Whether or not *Nitrospira* in RAS move nitrogen pools through these alternate pathways is not yet known.

Given the diversity of nitrifiers and burgeoning understanding of nitrifier metabolic flexibility, it is possible that some of the identified ammonia-oxidizing organisms in our system were not carrying out ammonia oxidation, as this scenario has been observed in municipal wastewater treatment systems (Mußmann et al., 2011). Our model indicates the measured ammonia removal could support the predicted ammonia-oxidizer biomass, and in fact overestimated the number of ammonia oxidizing cells present. This overestimation could be the result of the model's reliance on biomass production from traditional AOM metabolisms, which many not represent accurately biomass production from ammonia oxidation for metabolically flexible ammonia-oxidizers or comammox *Nitrospira* (Costa et al., 2006). Also, the cell volume used in the model is based on measurements of *Candidatus* Nitrosocosmicus franklandus, a relatively small microorganism; thus differences in cell size across ammonia-oxidizing taxa also may be contributing to the overestimation of biomass. In order to accurately predict ammonia consumption to biomass production ratios, which are used to constrain biofilter design, future models will need to account for the substrate kinetic differences between ammonia oxidizer metabolic pathways, differences in cell size among taxa, and include an updated understanding of cross-feeding between AOM and NOB (De Schryver and Vadstein, 2014; Daims et al., 2016).

This study builds upon the accumulating body of evidence that biofilter microbial communities in freshwater RAS are dynamic, diverse, and more distributed by resource availability than is often considered in the design process. Our results along with others (Sakami et al., 2012; Brown et al., 2013) indicate the microorganisms carrying out nitrification in RAS are different than those used traditionally to model RAS nitrifying capacity. This disconnect suggests there is potential to further fine-tune biofilter design to take advantage of these newly discovered physiologies and alter start-up procedures so that animal production objectives are matched to the nitrifying microorganisms most capable of meeting those demands. Incorporating this knowledge would provide opportunities to develop new system operations, such as operating at a lower pH (Hüpeden et al., 2016), and could move system optimization beyond that bound by current nitrification models. Yet, many unknowns remain, including how differences in system scale, water properties, and system initiation with subsequent founder effects influence biofilter community composition, stability, and ultimately performance. Further use of microbial ecological theory in aquaculture has the potential to extend RAS capabilities, identify currently unrecognized interactions between microorganisms and system design, and facilitate replicable zero discharge systems (De Schryver and Vadstein, 2014).

## Author contributions

RB contributed to the development of research project goals, carried out the lab work and most of the data analysis, and was the primary author in writing and revising the manuscript. SM was involved in writing and editing the manuscript and provided the primary source of funding. RN contributed to the development of research project goals, provided data analysis, was involved in all of the writing and editing of the manuscript, and contributed a source of project funding.

## Funding

Funding for this work was provided by a University of Wisconsin System Incentive grant to the School of Freshwater Sciences and through start-up laboratory funds to RN.

### Conflict of interest statement

The authors declare that the research was conducted in the absence of any commercial or financial relationships that could be construed as a potential conflict of interest.
