# Supplementary material for: Freshwater Recirculating Aquaculture System Operations Drive Biofilter Bacterial Community Shifts around a Stable Nitrifying Consortium of Ammonia-Oxidizing Archaea and Comammox Nitrospira
Source: Front Microbiol. 2017 Jan 30;8:101. doi: 10.3389/fmicb.2017.00101 (PMC5276851; doi:10.3389/fmicb.2017.00101)
Supplement: Supplementary file 3 [file Presentation1.PDF]

Supplementary Material

Freshwater Recirculating Aquaculture System Operations Drive Biofilter Bacterial Community Shifts Around a Stable Nitrifying Consortium of Ammonia-oxidizing *Archaea* and Comammox *Nitrospira*

Authors: Ryan P. Bartelme[1], Sandra L. McLellan[1], and Ryan J. Newton[1*]

* Correspondence: Ryan J. Newton, newtonr@uwm.edu

Table S1 - Sample metadata
See excel spreadsheet Table S1.

Table S2 – Genus level taxonomy for bacterial and archaeal biofilter sand and water sample sequences.
See attached excel file Table S2.

Table S3. qPCR assay statistics

Assay	Mean Slope[1]	R^2	Mean Efficiency (%)	Mean Y-int	Mean NTC Ct Value
Archaea 16S	-3.44	0.997	95.67	39.01	Undetected
Bacteria 16S	-3.61	0.997	89.38	36.79	34.18
Archaea *amoA*	-3.58	0.999	90.28	32.84	Undetected
UWM *nitroso*-1 *amoA* (*Nitrosomonas*)	-3.39	0.998	97.30	33.77	Undetected
UWM *nitroso-2 amoA* (*Nitrosomonas*)	-3.33	0.999	99.51	31.77	Undetected
uwm-1 *nxrB* (*Nitrospira*)	-3.47	0.998	94.15	36.69	Undetected
uwm-2 nxrB (*Nitrospira*)	-3.34	1.000	99.24	36.16	35.25
Comammox *Nitrospira amoA*	-3.32	0.998	99.97	33.56	Undetected

1. In all instances, mean refers to the mean slope, efficiency, and Y-intercept between repeated reactions.

2. Each new assay developed in this study was carried out with the protocols listed in the main text. The appropriate primer concentrations for the new assays were found by serially diluting DNA extracted from a sand sample of the surface of the UWM SFS RAS biofilter matrix. After determining the appropriate primer concentration, the annealing temperature was determined by running a temperature gradient on the full standard curve, one biofilter sand sample, and a no template control. Cross-reactivity was tested using a non-target genotype standard curve, sample, and no template control for assays where we differentiated between genotypes (*Nitrosomonas amoA* & *Nitrospira nxrB*). Assay annealing temperatures were adjusted to eliminate amplification of non-target templates. Results were validated by gel electrophoresis and melt curve analysis.

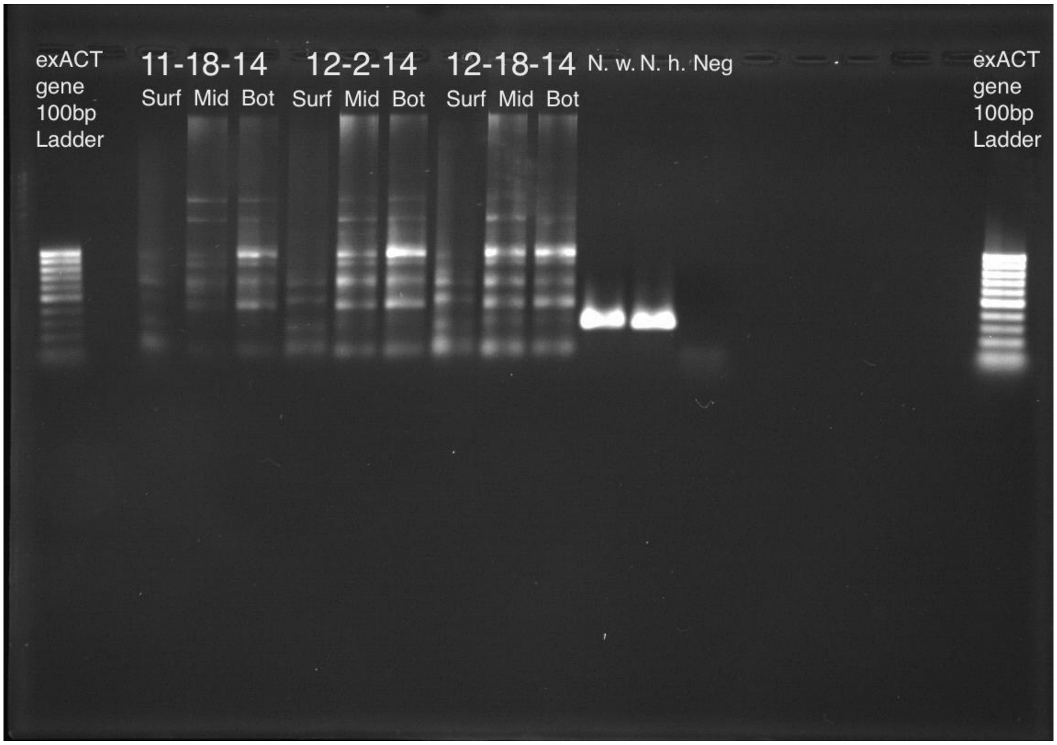

Figure S1 – *Nitrobacter* sp. *nxrA* PCR Gel. Amplified PCR-products from reactions using *nxrA* primers (1, 2) to target *Nitrobacter* spp. in biofilter sand samples from three timepoints and three depths. PCR-products are visualized in a 1% agarose gel stained with ethidium bromide. *N.w.* is *Nitrobacter winogradskyi* and *N. h.* is *Nitrobacter hamburgensis,* Neg=No template control*.* No strong PCR product is present and of the same size as the *Nitrobacter* spp. positive controls.

References

1. **Poly F**, **Wertz S**, **Brothier E**, **Degrange V**. 2008. First exploration of Nitrobacter diversity in soils by a PCR cloning-sequencing approach targeting functional gene nxrA. FEMS Microbiol. Ecol. **63**:132–140.
2. **Wertz S**, **Poly F**, **Le Roux X**, **Degrange V**. 2008. Development and application of a PCR-denaturing gradient gel electrophoresis tool to study the diversity of Nitrobacter-like nxrA sequences in soil. FEMS Microbiol. Ecol. **63**:261–271.
